# Four-Pyroptosis Gene-Based Nomogram as a Novel Strategy for Predicting the Effect of Immunotherapy in Hepatocellular Carcinoma

**DOI:** 10.1155/2022/2680110

**Published:** 2022-06-22

**Authors:** Ning Li, Shao-hua Ren, Ya-fei Qin, Bo Shao, Hong Qin, Zhaobo Wang, Hong-da Wang, Guang-ming Li, Yang-lin Zhu, Cheng-lu Sun, Jing-yi Zhang, Gang-gang Shi, Xing-wei An, Hao Wang

**Affiliations:** ^1^Department of Integrated Chinese and Western Medicine Hospital, Tianjin University, Tianjin, China; ^2^Department of General Surgery, Tianjin Medical University General Hospital, Tianjin, China; ^3^Tianjin General Surgery Institute, Tianjin Medical University General Hospital, Tianjin, China; ^4^Department of Anorectal Surgery, The Second Hospital of Tianjin Medical University, Tianjin, China; ^5^Academy of Medical Engineering and Translational Medicine, Tianjin University, Tianjin, China

## Abstract

**Background:**

Immunotherapy has been considered as a promising cancer treatment for hepatocellular carcinoma (HCC). However, due to the particular immune environment of the liver, identifying patients who could benefit from immunotherapy is critical in clinical practice.

**Methods:**

The pyroptosis gene expression database of 54 candidates from The Cancer Genome Atlas (TCGA) were collected to discover the critical prognostic-related pyroptosis genes. A novel pyroptosis gene model was established to calculate the risk score. Kaplan–Meier analysis and receiver operating characteristic curve (ROC) were used to verify its predictive ability. The International Cancer Genome Consortium (ICGC) data was collected as external validation data to verify the model's accuracy. We employed multiple bioinformatics tools and algorithms to evaluate the tumor immune microenvironment (TIME) and the response to immunotherapy.

**Results:**

Our study found that most pyroptosis genes were expressed differently in normal and tumor tissues and that their expression was associated with the prognosis. Then, a precise four-pyroptosis gene model was generated. The one-year area under the curves (AUCs) among the training, internal, and external validation patients were 0.901, 0.727, and 0.671, respectively. An analysis of survival data revealed that individuals had a worse prognosis than patients with low risk. The analysis of TIME revealed that the low-risk group had more antitumor cells, fewer immunosuppressive cells, stronger immune function, less immune checkpoint gene expression, and better immunotherapy response than the high-risk group. Immunophenoscore (IPS) analysis also demonstrated that the low-risk score was related to superior immune checkpoint inhibitors therapy.

**Conclusion:**

A nomogram based on the four-pyroptosis gene signature was a novel tool to predict the effectiveness of immunotherapy for HCC. Therefore, individualized treatment targeting the pyroptosis genes may influence TIME and play an essential role in improving the prognosis in HCC patients.

## 1. Introduction

Hepatocellular carcinoma (HCC) is the sixth most commonly diagnosed malignancy and the third leading cause of cancer mortality worldwide [1]. As a novel treatment, immunotherapy has represented an encouraging breakthrough for HCC patients [2]. Recently, various immune checkpoint blockade (ICB) therapeutic approaches have been employed successfully in treating HCC [3–5]. However, immunotherapy is not effective for all HCC patients [6–8]. At present, the poor response of some patients to immunotherapy is a thorny problem faced by clinicians. Specific signal molecules and stimulation that induce cell death can activate cells to produce different forms of immunogenic cell death (ICD). Dying tumor cells induce activation of antigen-presenting cells (APCs) through various immune signaling pathways, resulting in the antitumor immune response of cytotoxic T cells and natural killer cells (NK). Apoptosis resistance might be a key mechanism for tumor immune evasion [9]. Therefore, the induction of new forms of cell death may be a novel strategy to improve the efficacy of immunotherapy for HCC.

Pyroptosis is a typical programmed cell death (PCD) activated by caspase-1, characterized by the rupture of plasma membrane and release of proinflammatory substances [10]. Pyroptosis can cause a strong inflammatory reaction [11, 12]. A growing number of studies have indicated that pyroptosis genes were strongly linked to the occurrence and development of malignancies [13], including breast cancer [14], lung cancer [15], pancreatic ductal adenocarcinoma [16], and gastric cancer [17]. However, researchers have come to opposing conclusions in exploring the causal relationship among pyroptosis, prognosis, and immune microenvironment of HCC. According to a study, pyroptosis genes have been proved to be favorable for the survival of HCC [18]. According to the findings by Chu et al. [19], when pyroptosis is induced, inflammatory chemicals are produced, which limit the proliferation and migration of HCC cells while also enhance antitumor immunity. In contrast, another study found that pyroptosis aggravates inflammation, thereby leading to liver damage and the development of liver fibrosis and HCC [20]. Currently, the correlation between pyroptosis and HCC prognosis and tumor immune microenvironment (TIME) has not been fully elucidated.

A precise prediction of the pyroptosis gene risk may assist in regulating the immune microenvironment of HCC, allowing patients to receive appropriate treatment and improve their overall prognosis. Based on a four-pyroptosis gene model, we have developed a nomogram to distinguish cold and hot tumors and to predict the immunotherapy landscape in HCC. We will also explore the function and mechanism of cell death in the immunological microenvironment of liver cancer, thereby discovering the targets for new pharmacological therapy and immunotherapeutic techniques for HCC.

## 2. Material and Methods

### 2.1. HCC Patients' Data Acquisition, Collation, and Pyroptosis Gene Selection

The RNA-seq data and corresponding clinical information from 374 HCC patients and 50 healthy controls were downloaded from The Cancer Genome Atlas (TCGA). The 260 HCC patients from the International Cancer Genome Consortium (ICGC) cohort were obtained to validate our findings further. Fragments per kilobase million (FPKM) data were converted to transcripts per kilobase million (TPM) and normalized by dividing each value by the sum of all FPKM values for each tumor sample, followed by multiplication by 1 × 10^6^. In cases of more than one probe per gene, average values were chosen. This study has been approved by Tianjin Medical University General Hospital Human Ethics Committee. By retrieving the Gene Card database (https://www.genecards.org/) and consulting existing studies, we chose 54 pyroptosis candidate genes for analysis [20–23].

### 2.2. Differential Expression and Mutation Analysis of Pyroptosis Genes

The R package “limma” was used to analyze the differential expression of the 54 candidate pyroptosis genes between HCC and normal samples with false discovery rate (FDR) <0.05 and |log2 (fold change) |≥1.0. Simple nucleotide variation (SNP) data were acquired from the Genomic Data Commons Data Portal. The waterfall plots were constructed by the R package “maftools.”

### 2.3. Consensus Cluster Analysis of Pyroptosis-Related Subtypes

Using the R package “consensusClusterPlus,” we clustered the samples to establish multiple subgroups based on their candidate gene sets of pyroptosis genes with reps = 50, pItem = 0.8, and pFeature = 1 [24].

### 2.4. Functional and Pathway Enrichment Analysis of the Prognosis-Related Pyroptosis Genes

Gene Ontology (GO) and Kyoto Encyclopedia of Genes and Genomes (KEGG) were used to analyze the biological function and pathways of differentially expressed genes (DEGs) [25, 26].

### 2.5. Identification of the Pyroptosis Genes with Patients' Prognosis and Calculation of the Risk Score

In order to identify independent prognostic-related genes, least absolute shrinkage and selection operator (LASSO) regression and multivariate Cox regression analyses were conducted sequentially. The risk score was calculated using the following methodology:

(1) $$\begin{array}{c} \text{Risk score}=\overset{n}{\underset{i=1}{\sum }}\left(\text{Gene expression}*\text{Coefficient}\right) \end{array}$$

Patients were divided into two groups using the median risk score. The pyroptosis genes mode was tested using the Kaplan-Meier curve and receiver operating characteristic curve (ROC) drawn with R's “survival” and “timeROC” packages.

### 2.6. Construction of a Nomogram Based on the Four-Pyroptosis Gene Model, Internal and External Validation

A total of 165 patients were enrolled in the training cohort, and a nomogram was created using random resolution. The remaining 164 cases were utilized to verify the accuracy of the nomogram. The external validation data was obtained from the LIRI-JP dataset from the ICGC database. The 1-, 3-, and 5-year receiver operating characteristic (ROC) curves plotted with the R package “timeROC” were used to evaluate the predictive ability of the prognostic genes model for overall survival (OS). A nomogram was constructed with the R package “rms” and “regplot” to predict 1-, 3-, and 5-year OS of HCC patients. The ROC and calibration analyses were then performed to test the predictive capacity of the nomogram.

### 2.7. Gene Set Variation Analysis (GSVA), Enrichment and Visualization

For GSVA, we calculated the enrichment score for each sample in the gene set. Specifically, we first used gene expression profiling, using the method of Hanzelmann et al. [27], and downloaded the enrichment scores from the Molecular Signatures Database (MsigDB, http://www.gsea-msigdb.org/gsea/downloads.jsp) to evaluate the relevant pathways and molecular mechanisms. The final enrichment score matrix was obtained GSVA was implemented by the R package “h.all.v7.4.symbols”.

### 2.8. Correlation among the Immune Cell Infiltration, Tumor Immune Score, Immunotherapy-Related Gene Expression, Immunotherapy Responsiveness, and Risk Score

The tumor infiltration immune cells were subjected to the ESTIMATE [28], CIBERSORT [29], TIMER [30], CIBERSORT-ABS [29], QUANTISEQ [31], MCPCOUNTER [32], and xCell [33] analyses. The ESTIMATE algorithm calculated the immune and stromal score by the R package “estimate.” Analyses were conducted on the expression of immunotherapy-related genes between high- and low-risk groups. Furthermore, immunephenoscore (IPS) proved to be an excellent predictor of response to anti-CTLA-4 and anti-PD-1 treatments [34]. Accordingly, the Cancer Immunome Atlas (TCIA, https://tcia.at/home) database was used to predict the efficacy of immunotherapy.

### 2.9. Analysis of NCI-60 Drug Database with CellMiner

Using the CellMiner database, we examined the impact of pyroptosis genes on drug sensitivity and tolerance. CellMiner (https://discover.nci.nih.gov/cellminer/) was developed by the National Cancer Institute (NCI) to integrate the molecular and pharmacological datasets for the NCI-60 cell line panel [35, 36]. In addition, the relationship between gene expression and drug sensitivity was analyzed by the Pearson correlation test.

### 2.10. Statistical Analysis

All the analyses were performed by R software (version 4.1.0). The Kaplan-Meier survival analysis was generated by the R package “survival” and compared with the log-rank test. The chi-square test was used for correlation analysis. The infiltration of immune cells was compared by the Wilcox Test. All tests were bilateral, and *p* < 0.05 was statistically significant.

## 3. Results

### 3.1. The Data for HCC Patients

**Figure 1:**
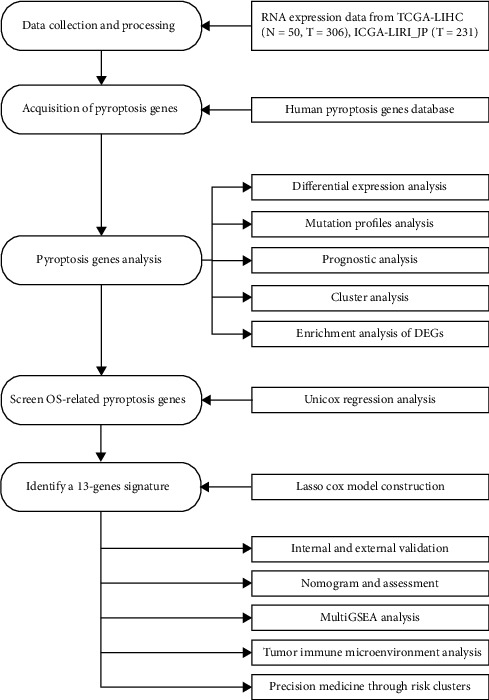
Diagram summarizing the workflow of this study from data collection and processing immune analysis.

After eliminating cases with incomplete survival data, the mRNA expression and clinical data of 329 individuals were obtained (Figure 1). 231 HCC samples from the ICGC-LIRI-JP dataset were obtained to further validate our findings. In addition, the detailed characteristics of all patients were shown in Table 1.

### 3.2. Differential Expression Analysis and Mutation Analysis of the 54 Candidate Pyroptosis Genes

**Figure 2:**
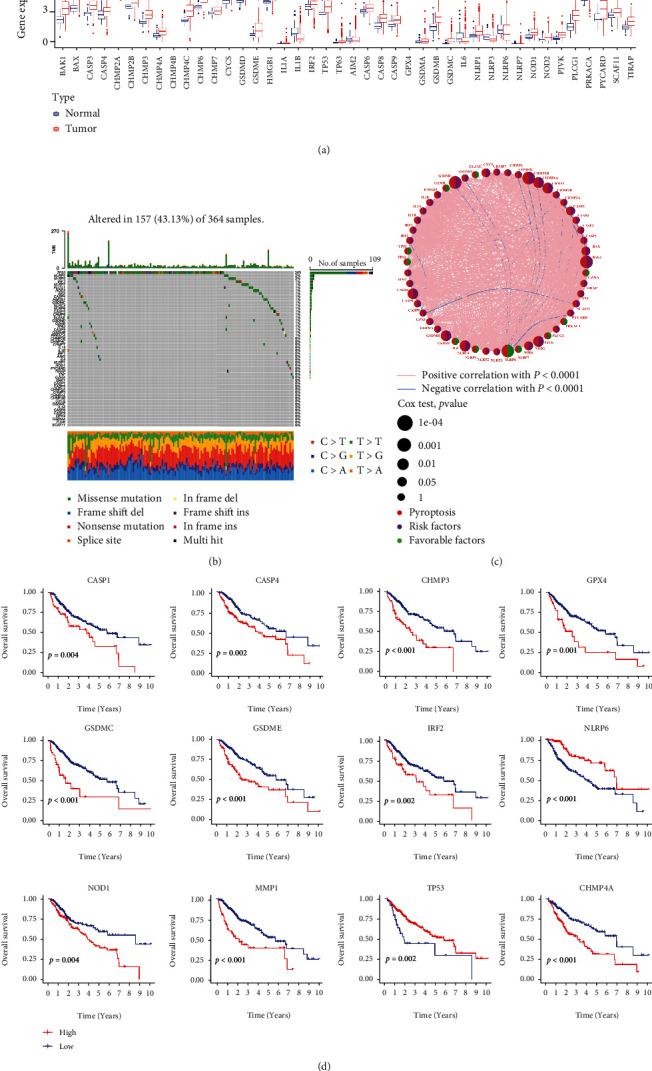
Expressions and mutation of the pyroptosis genes and their relationship with prognosis. (a) Gene expression distributions of pyroptosis genes in normal and tumor samples from TCGA databases; (b) somatic mutation waterfall plot of the pyroptosis genes; (c) the correlation network of the pyroptosis-related genes; (d) identification of the OS-related pyroptosis genes in the TCGA cohort with KM analysis. ns: not significant, ∗*p* < 0.05, ∗∗*p* < 0.01, and ∗∗∗*p* < 0.001.

On the basis of the TCGA database, 42 out of 54 candidate pyroptosis genes were different between tumor tissues (*n* = 374) and normal tissues (*n* = 50). The maftools package indicated the differences in distributions of somatic mutations of the pyroptosis genes (Figure 2(b)). A comprehensive landscape correlation network based on the univariate Cox regression analysis and the Kaplan–Meier survival analysis was constructed to better understand the interaction between pyroptosis genes and the prognosis of HCC (Figures 2(c) and 2(d)). These results demonstrated that pyroptosis genes were involved in the genesis and progression of tumors.

### 3.3. Consensus Cluster Analysis of Pyroptosis-Related Subtypes

**Figure 3:**
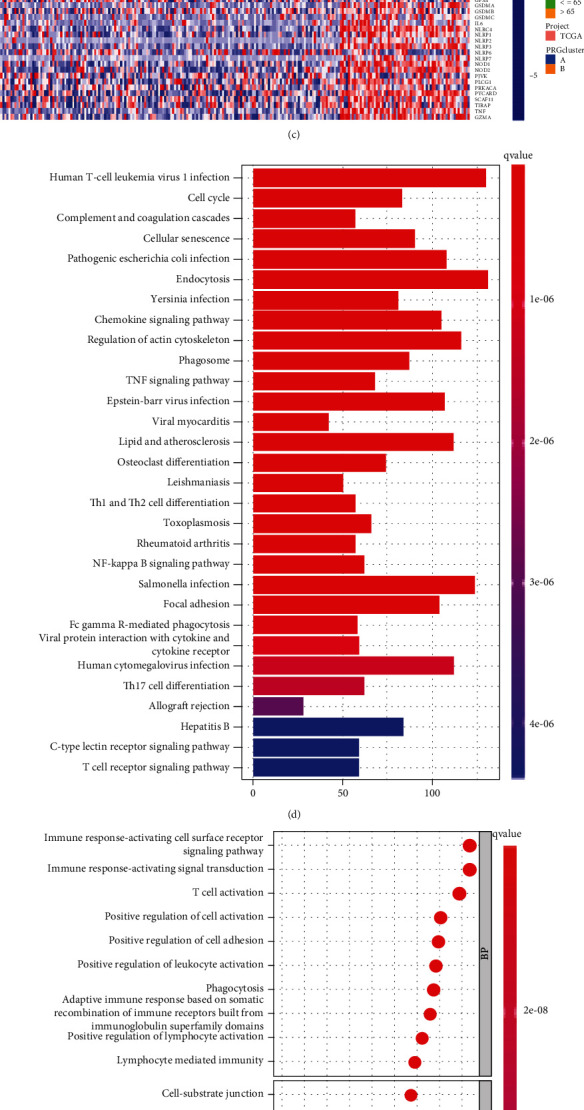
Identification of potential subtypes of HCC based on pyroptosis genes. (a) The samples were stratified into 2 clusters based on the consensus clustering matrix (*k* = 2); (b) survival analysis of pyroptosis clusters A and B; (c) the relationship between tumor stage, grade, gender, age, and pyroptosis clusters; (d, e) KEGG and GO analysis of DEGs between pyroptosis clusters A and B.

Based on the expression of prognosis-related pyroptosis genes, we then created unsupervised consensus clusters. It was determined that *k* = 2 (Figure 3(a)) had the highest clustering stability from *k* = 2 to 9 (Supplementary Figure 1a-1k). However, in further analysis, we found that pyroptosis-related subtypes were substantially connected with the OS of HCC, not with clinicopathological indicators (Figures 3(b) and 3(c)). This result suggests that the HCC prognostic criteria should be classified more precisely.

### 3.4. Functional and Pathway Enrichment Analysis of the Prognosis-Related Pyroptosis Genes

KEGG analysis showed that the DEGs mainly were abundant in immune regulation, cell cycle, cellular senescence, and endocytosis (Figure 3(d)). The functional annotation results revealed that these prognosis-related pyroptosis genes were mainly associated with biological processes such as immune response, immune regulation, cell activation, and cell adhesion (Figure 3(e)).

### 3.5. Calculation of the Risk Score and the Correlation among the Pyroptosis Genes, the Clinicopathological Characteristics, and the Risk Score

**Figure 4:**
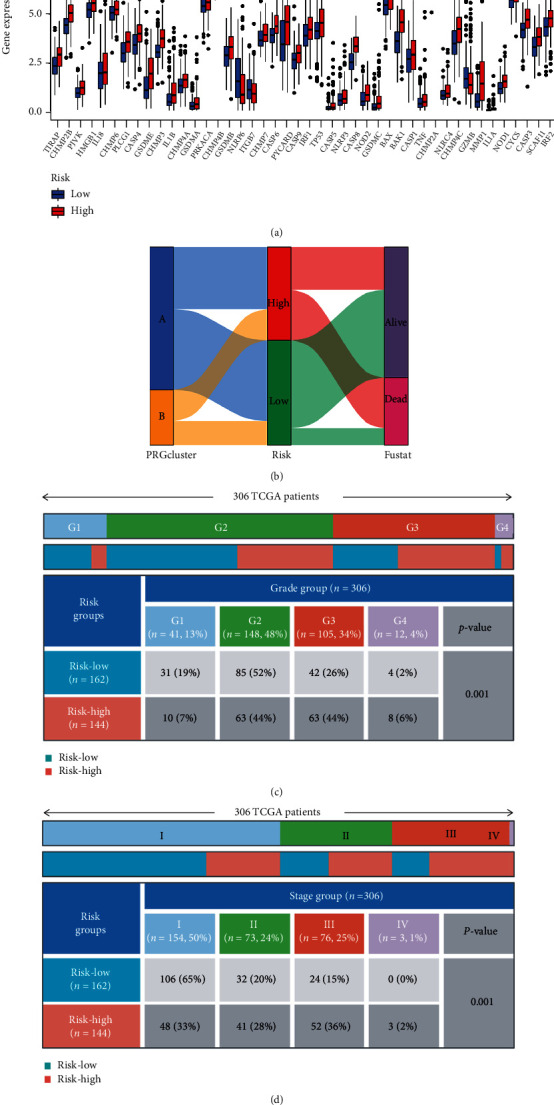
Clinical verification of pyroptosis scores. (a) Pyroptosis genes expression distributions in the high- and low-risk groups; (b) the Sankey diagram shows the flow diagram of our investigation; (c, d) the relationship between grade and tumor stage of this novel signature clinical characteristics. ns: not significant, ∗*p* < 0.05, ∗∗*p* < 0.01, and ∗∗∗*p* < 0.001.

The 54 candidate pyroptosis genes were included in the Lasso-Cox multivariate analysis (Supplementary Figures 2a and 2b). Finally, four prognosis-related pyroptosis genes were identified as the independent risk factors (Table 2). Accordingly, the risk score of each patient was calculated based on the expression of each independent prognosis-related pyroptosis gene and its coefficient. The median risk score was employed to separate all the patients into high-risk and low-risk groups. The expression of pyroptosis genes showed a substantial difference in the high- and low-risk groups (Figure 4(a), *p* < 0.05). The pyroptosis-related subtypes, risk score groups, and future states are summarized in a Sankey diagram (Figure 4(b)). There were also significant differences in disease stage and pathological grade in the high- and low-risk groups (Figures 4(c) and 4(d), *p* = 0.001 and *p* = 0.001). High-risk scores were strongly associated with higher disease stage and pathological grade.

### 3.6. Identification of the Prognosis Model, Internal and External Verification of the Four Pyroptosis Genes Model, and Construction of a Nomogram

**Figure 5:**
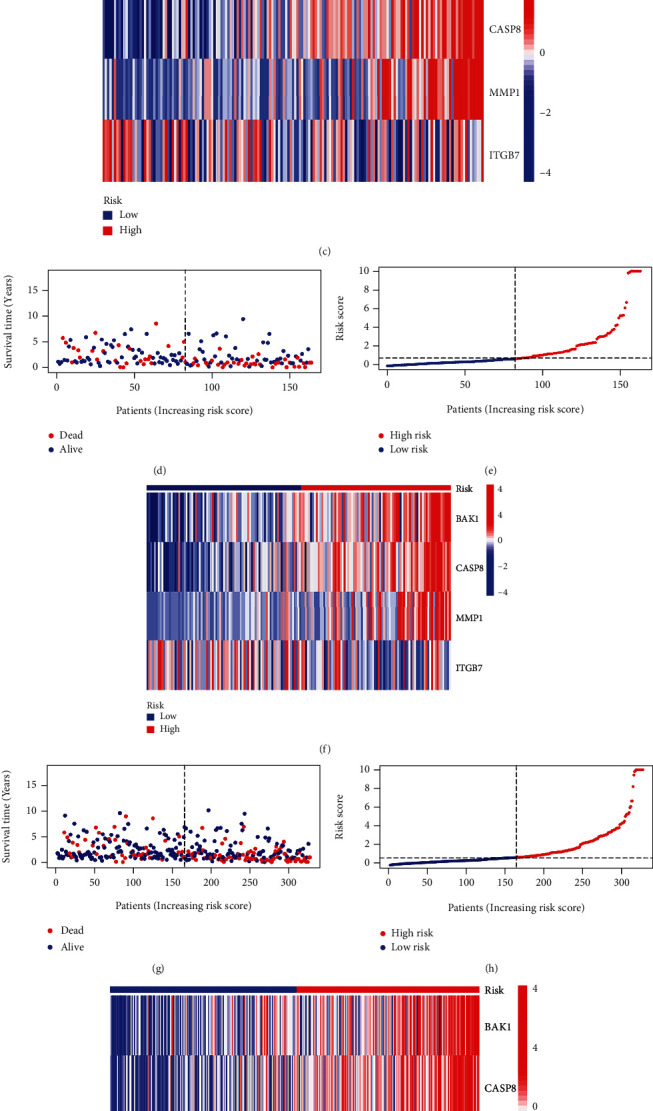
(a–c) Distribution of the risk scores, survival status, and expression of the four pyroptosis genes model in the training cohort. (d–f) Distribution of the risk scores, survival status, and expression of the four pyroptosis genes model in the internal validation cohort. (g–i) Distribution of the risk scores, survival status, and expression of the 13 pyroptosis genes model in all TCGA patients.

**Figure 6:**
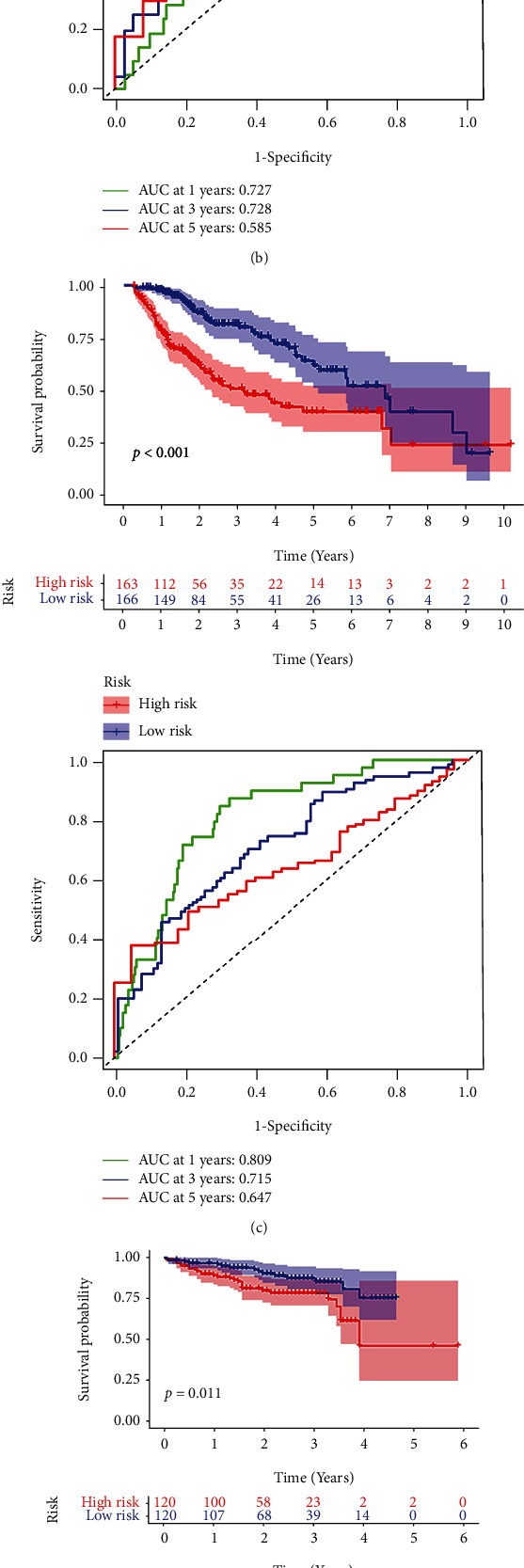
Construction of pyroptosis risk signature. (a) ROC curve and Kaplan-Meier survival curve of the model based on data from the training cohort; (b) ROC curve and Kaplan-Meier survival curve of the model based on data from the internal validation cohort; (c) ROC curve and Kaplan-Meier survival curve of the model based on all TAGA-LIHC patients' data; (d, e) further verification of the risk classification of HCC patients using external ICGC-LIRI-JP data; (f) ROC curves for predicting survival rates of HCC patients with the pyroptosis risk score, age, gender, grade, and tumor stage; (g) developed nomogram based on the pyroptosis risk score and clinicopathological parameters; (h) calibration curve of the 1-year, 3-year, and 5-year survival rates of the model.

The distribution of risk scores and survival status showed patients with higher risk scores in the training cohort (Figures 5(a) and 5(b)) and the internal validation cohort (Figures 5(d) and 5(e)), and all patients (Figures 5(g) and 5(h)) showed a higher probability of death. In addition, the relationships between the four independent prognosis-related pyroptosis genes and the risk score were shown in heat maps (Figures 5(c), 5(f), and 5(i)). Among the training cohort (Figure 6(a)) and the internal validation cohort (Figure 6(b)), as well as the entire patient group (Figure 6(c)), we found that high-risk patients have a worse prognosis than patients with a low risk (*p* < 0.001, *p* = 0.014, and *p* < 0.001). The area under the curve (AUC) of the training cohort, the internal validation cohort, and the entire patient group at 1, 3, and 5 years were 0.901, 0.727, and 0.809; 0.720, 0.728, and 0.715; and 0.738, 0.585, and 0.647, respectively (Figures 6(a)–6(c)). Using the same coefficient, the risk score of the LIRI-JP dataset was calculated and grouped by the median score. OS was better for low-risk patients than high-risk patients in the verification group based on the survival curve (Figure 6(d), *p* = 0.011). The AUC at 1, 3, and 5 years were 0.671, 0.633, and 0.356 (Figure 6(e)). The ROC curve showed a better area AUC achieved with the risk score than the other clinicopathological characteristics (Figure 6(f)). Then, we constructed a nomogram based on the risk score, gender, age, grade, and stage to predict the prognosis of HCC patients (Figure 6(g)). The calibration plot showed that patients' 1, 3, and 5 years of survival were consistent between the predicted and observed values (Figure 6(h)). To make the clinic application of the model more convenient, we developed a web-based nomogram (Supplementary Figure 3).

### 3.7. Gene Set Variation Analysis (GSVA), Enrichment and Visualization

**Figure 7:**
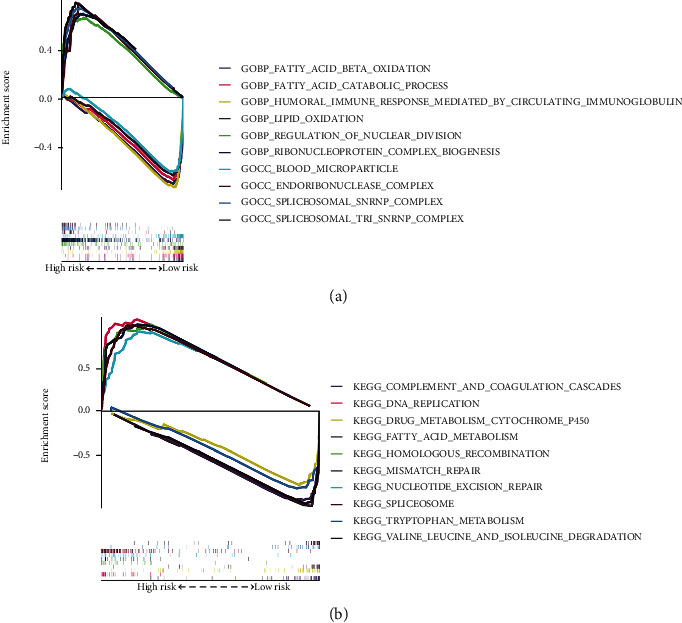
GSEA of the significantly enriched GO (a) and KEGG (b) pathways in the TCGA cohort classified by the high- and low-risk groups.

Furthermore, we have found that the GSEA analysis significantly enriched the immune-related signaling pathways, fatty acid metabolism, and genetic regulation (Figures 7(a) and 7(b)).

### 3.8. Correlation among the Immune Cell Infiltration, Tumor Immune Score, Immunotherapy-Related Genes Expression, Immunotherapy Responsiveness, and Risk Score

**Figure 8:**
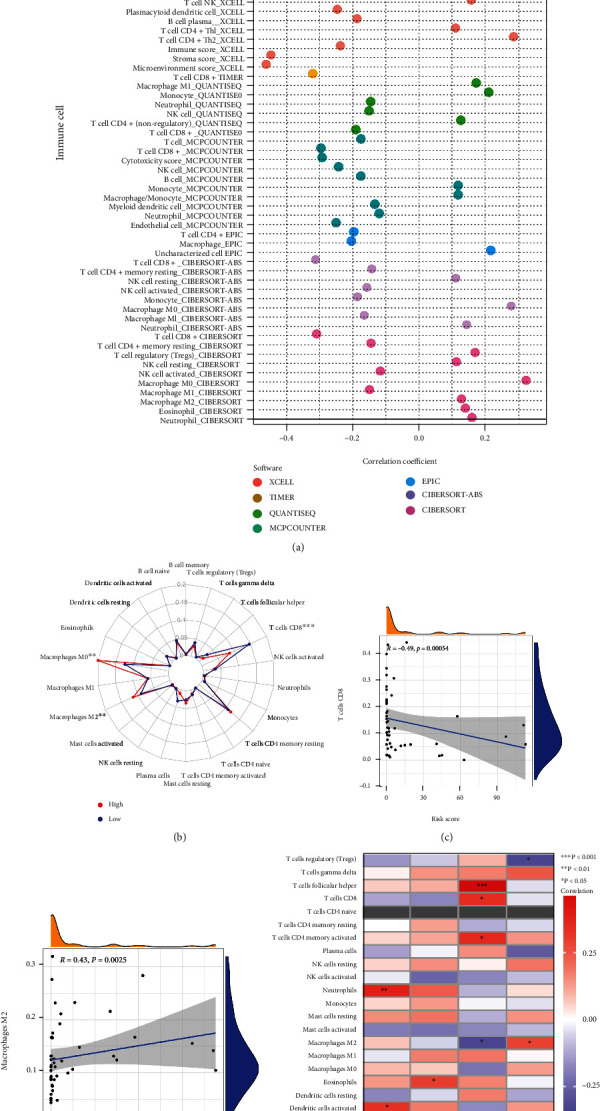
Pyroptosis risk scores in the prediction of immunotherapy. (a) The correlation analysis of the immune infiltration and the risk scores; (b) radar plot showing cell infiltration from the CIBERSORT procedure; (c) the correlation analysis of the CD8^+^ T cells and the risk scores; (d) the correlation analysis of the M2 macrophages and the risk scores; (e) the correlation analysis of the immune infiltration and the 13 independent prognostic pyroptosis genes. ns: not significant, ∗*p* < 0.05, ∗∗*p* < 0.01, and ∗∗∗*p* < 0.001.

Our subsequent discussion explored the possible relationship between risk score and immunotherapy efficacy by examining the correlation among immune cell infiltration, immunotherapy-related genes expression, tumor immune score, and risk score. First, we performed derivations using multiple bioinformatics tools and algorithms, which showed that immune cells were significantly associated with risk scores (Figure 8(a)). A higher number of immunosuppressive cells such as M2 and M0 macrophages were found in the high-risk group (Figure 8(b), *p* < 0.01), while a higher number of antitumor immune cells such as CD8^+^T cells were found in the low-risk group (Figure 8(b), *p* < 0.001). Moreover, we found that the risk score was significantly and negatively correlated with the CD8^+^T cells and the predominant antitumor cells within the TIME (Figure 8(c), *p* = 0.00054 and *r* = −0.49). On the other hand, M2 macrophages were significantly and positively correlated with the risk score (Figure 8(d), *p* = 0.0025 and *r* = 0.43). Tumor-infiltrating immune cells were closely related to the four prognosis-related pyroptosis genes (Figure 8(e)).

**Figure 9:**
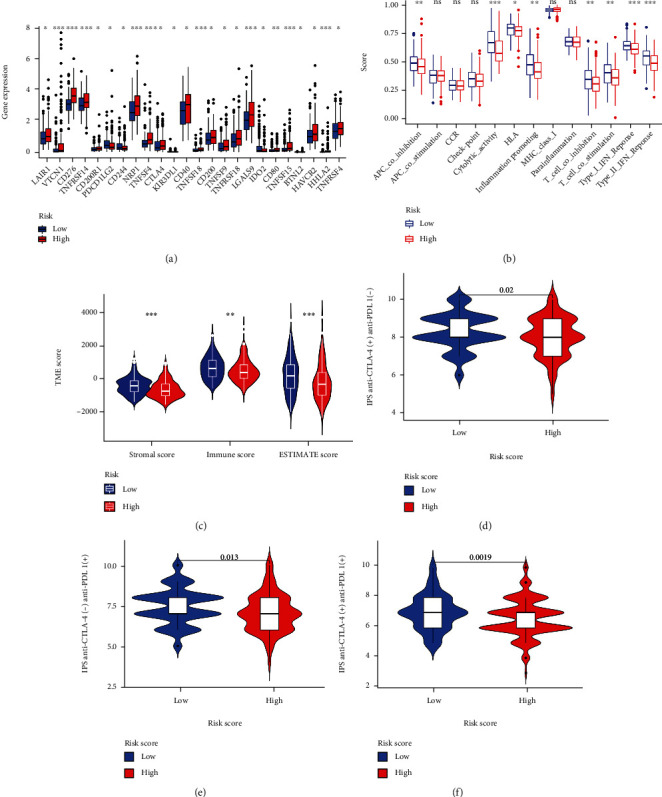
Immune-related functions and immunotherapy responsiveness of the high- and low-risk groups based on TCGA-LIHC cohort data. (a) Immune checkpoint gene expression of high- and low-risk groups; (b) the comparison of ssGSEA scores derived from 16 different immune cells and 13 immune signal pathways of high and low-risk groups; (c) the comparison of immune scores, stromal scores, and ESTAMETE scores of high- and low-risk groups. The potential effect of anti-CTLA4 (d), anti-PD-1 (e) and combination of two drugs (f) of the high- and low-risk groups based on the Cancer Immunome Atlas database (TCIA, https://tcia.at/home). ns: not significant, ∗*p* < 0.05, ∗∗*p* < 0.01, and ∗∗∗*p* < 0.001.

As compared with the low-risk group, the expression levels of immune checkpoint genes were significantly higher in the high-risk group (Figure 9(a)). Further analysis of infiltrating immune cells revealed that the functional score was significantly higher in the low-risk group than in high-risk groups (Figure 9(b)). Furthermore, the immune and ESTIMATE scores were significantly lower in the high-risk group than in the low-risk group (Figure 9(c)). In the low-risk group, treatment with anti-CTLA4 and anti-PD-1, either in monotherapy or combination therapy, was also more likely to affect HCC patients (Figures 9(d)–9(f), *p* = 0.02, *p* = 0.0013, and *p* = 0.0019). The pyroptosis genes risk score allowed us to divide HCC patients into two groups with distinct immune profiles. The low-risk group had more antitumor cells, fewer immunosuppressive cells, stronger immune function, less immune checkpoint gene expression, and better immunotherapy response than the high-risk group.

### 3.9. Analysis of NCI-60 Drug Database with CellMiner

**Figure 10:**
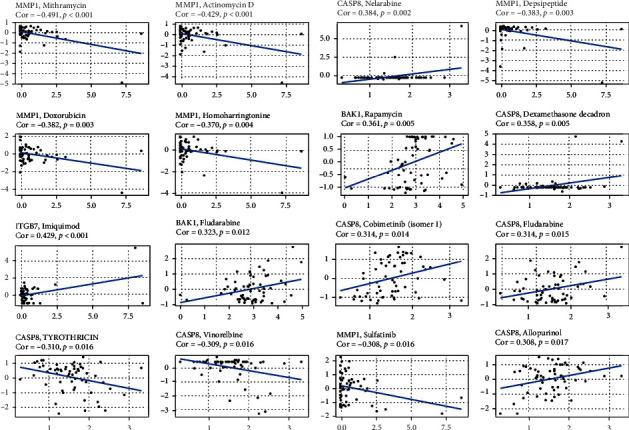
Sensitivity correlation analysis of independent prognostic pyroptosis genes and drugs based on the CellMiner Database.

To identify the top 16 drugs with the greatest significant differences, we conducted a separate drug sensitivity analysis based on the pyroptosis-related signature in the prognostic model (Figure 10). The results showed that the expression of MMP1 was negatively correlated with the sensitivity of Mithramycin (*r* = −0.491 and *p* < 0.001), Actinomycin D (*r* = −0.429 and *p* < 0.001), Depsipeptide (*r* = −0.383 and *p* = 0.003), Doxorubicin (*r* = −0.382 and *p* = 0.003), Homoharringtonine (*r* = −0.370 and *p* = 0.004), and Sulfatinib (*r* = −0.308 and *p* = 0.016). The expression of CASP8 was positively correlated with the sensitivity of Nelarabine (*r* = 0.384 and *p* = 0.002), Dexamethasone Decadron (*r* = 0.358 and *p* = 0.005), Cobimetinib (isomer 1) (*r* = 0.314 and *p* = 0.014), Fludarabine (*r* = 0.314 and *p* = 0.015), and Allopurinol (*r* = 0.308 and *p* = 0.017), but it was negatively correlated with the sensitivity of Tyrothricin (*r* = −0.310 and *p* = 0.016). The expression of BAK1 was positively correlated with the sensitivity of Rapamycin (*r* = 0.361 and *p* = 0.005) and Fludarabine (*r* = 0.323 and *p* = 0.012). The expression of ITGB7 was positively correlated with the sensitivity of Imiquimod (*r* = 0.429 and *p* < 0.001).

## 4. Discussion

The immune system plays a vital role in tumorigenesis. The liver is the largest immune-related organ, and liver cancer is subjected to a complicated immune microenvironment [37, 38]. In HCC, not all patients obtain the desired objective response rate after immunotherapy due to the heterogeneity of HCC [39]. As a result, reliable ways for identifying persons who could benefit from immunotherapy are urgently needed. Our findings preliminarily prove the potential role of cell death in the occurrence and development of HCC and the feasibility of using pyroptosis genes to construct a prognostic model.

Pyroptosis plays a vital role in biological development, dynamic balance, and cancer pathogenesis [40]. The majority of pyroptosis genes were shown to be differently expressed in normal and malignant tissues and substantially linked with the prognosis of HCC patients in this investigation. Then, using a four-pyroptosis gene model, we created a nomogram that can predict the immunotherapy landscape in HCC. This prediction model delivered excellent results in both the internal and external validation cohorts and was successfully verified.

Pyroptosis is a double-edged sword. A complex interaction between pyroptosis and cancer might have a function in tumor immunity in a different way. On the one hand, pyroptosis can stimulate tumor development by altering the tumor microenvironment. [22]. A growing number of investigations have shown that pyroptosis can promote the immune evasion of tumor cells by interfering with the immune microenvironment. Luan and Ju discovered that activated caspase-1 could stimulate hepatoma cells to pyroptosis, release proinflammatory cytokines, and further promote the growth of HCC [41]. In addition, NLRP3 can inhibit the antitumor immune response of gastric cancer by activating cell death [42]. Interestingly, the study discovered that cell death-induced inflammation can trigger antitumor immunity and has a synergistic effect with anti-PD1 treatment [43]. Previous reports indicate that pyroptosis is closely related to the efficacy of immunotherapy [44]. When tumor cells undergo pyroptosis, they could recruit tumor-suppressed immune cells and boost antitumor immunity. A study has shown that CD8^+^T cells can induce pyroptosis by releasing GzmA (GSDMB-cleaving enzyme) and GzmB (GSDME-cleaving enzyme) and further activating IL-1*β* from macrophages to play an antitumor effect [45]. Our immune analysis found that the four-pyroptosis gene model can reasonably predict the immunotherapy landscape in HCC. Patients were split into high- and low-risk groups based on pyroptosis genes risk score. In comparison to high-risk group, the low-risk group had more antitumor cells, fewer immunosuppressive cells, stronger immune function, and less immune checkpoint gene expression. These findings showed that a suppressed state of antitumor immunity may contribute to the poor outcome of high-risk individuals. In addition, the high-risk group exhibited a lower response to immunotherapy due to immunological dysfunction and the absence of infiltrating immune cells. These results suggest that scorched death genes influence the malignancy of tumors by altering the TIME. Low-risk patients may be more amenable to immunotherapy.

Pyroptosis is considered to be a promising direction in tumor therapy. Intervention against pyroptosis may be a novel and effective option for treating cancer. It has been found that small molecular drugs such as berberine, euxanthone, and miltirone may induce hepatoma cell death by activating cell pyroptosis [19, 46, 47]. Jiang et al. have found that metformin triggers the NF-*κ*B signal pathway, which leads to caspase-3/GSDME-mediated cancer cell death [48]. Many recent attempts to increase effectiveness by combining TACE and targeted treatment with immunotherapy have been documented [49–52]. As a local chemotherapy modality, TACE can promote better immunotherapy by modulating the TIME [50]. Several sensitive drugs were proven to target the four genes associated with pyroptosis. The results may also provide guidance for future chemotherapy, targeted therapy, and induced pyroptosis therapy combined with immunotherapy regimens for HCC.

To provide tailored therapy and enhance patients' long-term prognosis, precision medicine necessitates reliable prognosis evaluation. Our study may inform the future clinical treatment of HCC. However, there are certain shortcomings in this study. In order to better assess the accuracy and effectiveness of this model, it must be compared to a real-world dataset. Currently, the molecular mechanism between the genes identified by this signature and tumor immunity in HCC is still not clear, and further in-depth studies are warranted.

## 5. Conclusion

A nomogram based on the four-pyroptosis gene signature was a novel tool to predict the effectiveness of immunotherapy for HCC. Therefore, individualized treatment targeting the pyroptosis genes may influence TIME and play an essential role in improving the prognosis of the HCC patients.

## Figures and Tables

**Table 1:** Clinical information of the patients included in this study.

Characteristic	TCGA-LIHC (*N* = 306)	ICGC-LIRI-JP (*N* = 231)
Age (years)		
> 65 (%)	103 (33.66)	142 (61.47)
≤ 65 (%)	203 (66.34)	89 (38.53)
Sex		
Female (%)	94 (30.72)	61 (26.41)
Male (%)	212 (69.28)	170 (73.59)
Historical grade		
G1 (%)	41 (13.40)	—
G2 (%)	148 (48.37)	—
G3 (%)	105 (34.31)	—
G4 (%)	12 (3.92)	—
Stage		
I (%)	154 (50.33)	36 (15.58)
II (%)	73 (23.86)	105 (45.45)
III (%)	76 (24.84)	71 (30.74)
IV (%)	3 (0.98)	19 (8.23)

**Table 2:** The risk regression coefficients of four pyroptosis genes for establishing the risk score.

Ensembl ID	Symbol ID	Gene name	Coef
ENSG00000064012	CASP8	Caspase-8	0.861677
ENSG00000030110	BAK1	BCL2 antagonist/killer 1	0.354883
ENSG00000139626	ITGB7	Integrin subunit beta 7	-0.753754
ENSG00000196611	MMP1	Matrix metallopeptidase 1	0.353144

## Data Availability

The RNA-seq data, mutation data, and corresponding clinical information used to support the findings of this study have been deposited in The Cancer Genome Atlas (TCGA, https://portal.gdc.cancer.gov/) and International Cancer Genome Consortium (ICGC, https://dcc.icgc.org/) repository. The processed data are available from the corresponding author upon request.

## Supplementary Materials

Supplementary Materials Supplementary Figure 1: showed the identification of potential subtypes of HCC based on pyroptosis genes: (a) cumulative distribution function (CDF) curve, (b) cumulative delta area under CDF for the optimum decision of k value, (c) tracking plot, and (d–k) sample clustering heat map (*k* = 3 − 9). Supplementary Figure 2: showed the selection of factors for multivariate Cox regression analysis by LASSO regression analysis. Supplementary Figure 3: showed the Web-based dynamic (https://nomorsh.shinyapps.io/pyroptosis/).
