# Supplementary material for: Four-Pyroptosis Gene-Based Nomogram as a Novel Strategy for Predicting the Effect of Immunotherapy in Hepatocellular Carcinoma
Source: Biomed Res Int. 2022 Jun 22;2022:2680110. doi: 10.1155/2022/2680110 (PMC9242783; doi:10.1155/2022/2680110)
Supplement: Supplementary Materials — Supplementary Figure 1: showed the identification of potential subtypes of HCC based on pyroptosis genes: (a) cumulative distribution function (CDF) curve, (b) cumulative delta area under CDF for the optimum decision of k value, (c) tracking plot, and (d–k) sample clustering heat map (k = 3 − 9). Supplementary Figure 2: showed the selection of factors for multivariate Cox regression analysis by LASSO regression analysis. Supplementary Figure 3: showed the Web-based dynamic (https://nomorsh.shinyapps.io/pyroptosis/). [file 2680110.f1.zip › 2680110.f1/S3.pdf]

a

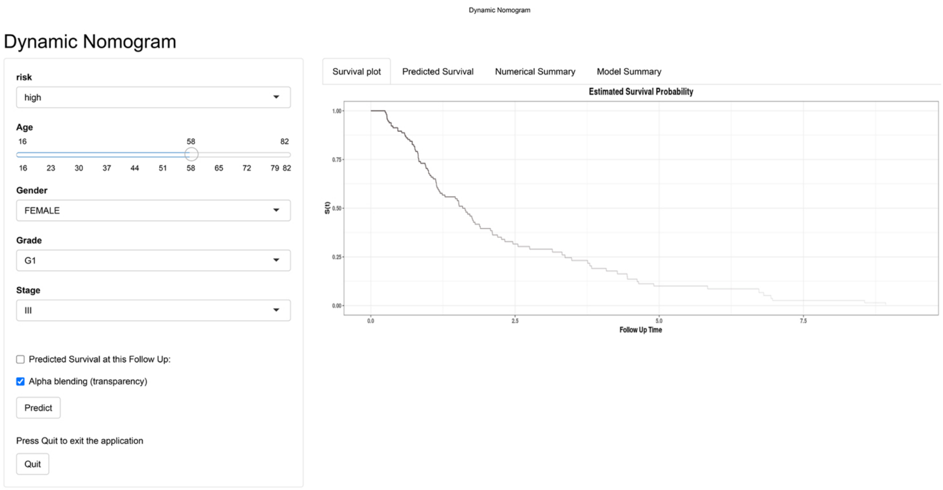
Dynamic Nomogram
Dynamic Nomogram
risk
high
Age
16
58
82
16 23 30 37 44 51 58 65 72 79 82
Gender
FEMALE
Grade
G1
Stage
III
Predicted Survival at this Follow Up:
Alpha blending (transparency)
Predict
Press Quit to exit the application
Quit
Survival plot
Predicted Survival
Numerical Summary
Model Summary
Estimated Survival Probability
S(t)
Follow Up Time

b

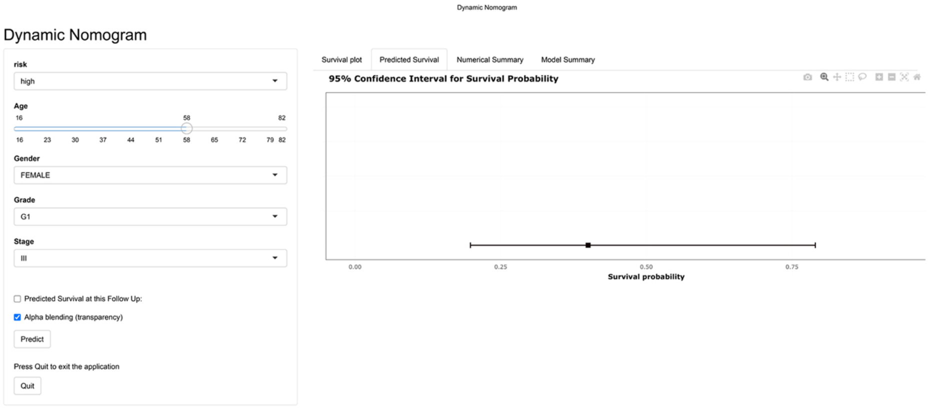
Dynamic Nomogram
Dynamic Nomogram
risk
high
Age
16
58
82
16 23 30 37 44 51 58 65 72 79 82
Gender
FEMALE
Grade
G1
Stage
III
Predicted Survival at this Follow Up:
Alpha blending (transparency)
Predict
Press Quit to exit the application
Quit
Survival plot
Predicted Survival
Numerical Summary
Model Summary
95% Confidence Interval for Survival Probability
0.00
0.25
0.50
0.75
Survival probability

c

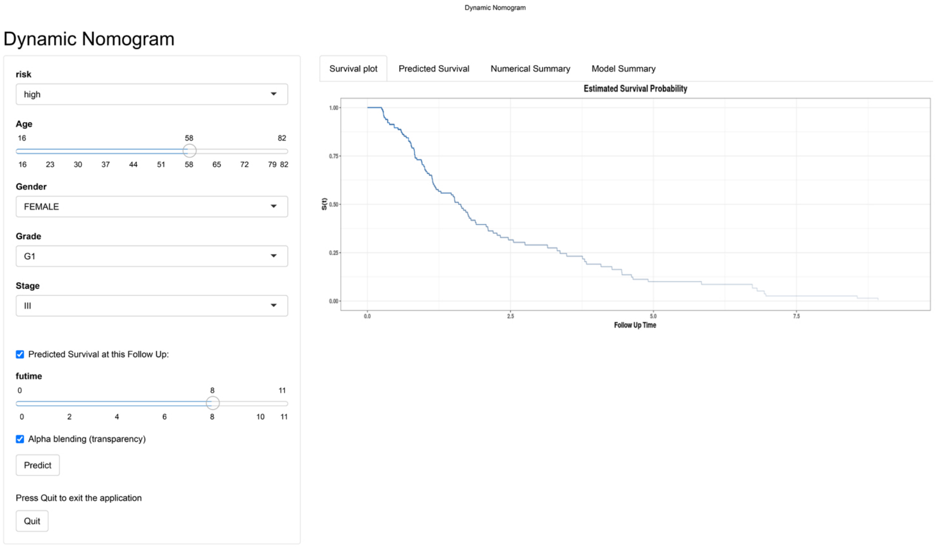
Dynamic Nomogram
Dynamic Nomogram
risk
high
Age
16
58
82
16 23 30 37 44 51 58 65 72 79 82
Gender
FEMALE
Grade
G1
Stage
III
Predicted Survival at this Follow Up:
futime
0
8
11
0 2 4 6 8 10 11
Alpha blending (transparency)
Predict
Press Quit to exit the application
Quit
Survival plot
Predicted Survival
Numerical Summary
Model Summary
Estimated Survival Probability
S(t)
Follow Up Time
