# Supplementary material for: Four-Pyroptosis Gene-Based Nomogram as a Novel Strategy for Predicting the Effect of Immunotherapy in Hepatocellular Carcinoma
Source: Biomed Res Int. 2022 Jun 22;2022:2680110. doi: 10.1155/2022/2680110 (PMC9242783; doi:10.1155/2022/2680110)
Supplement: Supplementary Materials — Supplementary Figure 1: showed the identification of potential subtypes of HCC based on pyroptosis genes: (a) cumulative distribution function (CDF) curve, (b) cumulative delta area under CDF for the optimum decision of k value, (c) tracking plot, and (d–k) sample clustering heat map (k = 3 − 9). Supplementary Figure 2: showed the selection of factors for multivariate Cox regression analysis by LASSO regression analysis. Supplementary Figure 3: showed the Web-based dynamic (https://nomorsh.shinyapps.io/pyroptosis/). [file 2680110.f1.zip › 2680110.f1/Supplementary Figure Legends.docx]

**Supplementary Figure Legends**

Supplementary Figure 1: Identification of potential subtypes of HCC based on pyroptosis genes. (a) cumulative distribution function (CDF) curve, (b) cumulative delta area under CDF for the optimum decision of k value, (c) tracking plot, and (d-k) sample clustering heat map (k = 3-9).

Supplementary Figure 2: (a) and (b) Selection of factors for multivariate Cox regression analysis by LASSO regression analysis.

Supplementary Figure 3: (a-c) Web-based dynamic (https://nomorsh.shinyapps.io/pyroptosis/).
